# The Use of CAD/CAM Technology in Mandibular Canine Disimpaction: A Case Report

**DOI:** 10.3390/dj12030079

**Published:** 2024-03-20

**Authors:** Francesca Germanò, Rosanna Guarnieri, Martina Mezio, Ersilia Barbato, Michele Cassetta

**Affiliations:** Department of Oral and Maxillofacial Sciences, School of Dentistry, “Sapienza” University of Rome, 00161 Rome, Italy; rosanna.guarnieri@uniroma1.it (R.G.); martina.mezio@uniroma1.it (M.M.); ersilia.barbato@uniroma1.it (E.B.); michele.cassetta@uniroma1.it (M.C.)

**Keywords:** CAD/CAM, surgical guide, miniscrew, impacted mandibular canine, digital orthodontics, TADs

## Abstract

This case report of an 11-year-old subject shows the digital workflow for the management of an impacted mandibular canine using Computer-Aided Design/Computer-Aided Manufacturing (CAD/CAM) technology along with Temporary Anchorage Devices (TADs). The miniscrew insertion site was planned using software, and a surgical guide was digitally designed and 3D printed. Orthodontic traction was performed using a 3D-designed and -printed device. In a single sitting, the miniscrew was inserted and the disimpaction device was also delivered. The primary objective of recovery and the repositioning of the impacted mandibular canine in the axis with its eruptive path was achieved. The space available and the subject’s early stage of mixed dentition was considered favourable to a spontaneous eruption. This case report shows how CAD/CAM digital technology, combined with 3D printing, enables the creation of a surgical guide to position the miniscrew and the customized devices used for mandibular canine disimpaction. CAD/CAM surgical guides can help clinicians to position TADs with more accuracy and predictability, ensuring high quality bone support offering primary stability. Although orthodontic traction is the most complex therapeutic choice to implement, with the aid of CAD/CAM technology it is possible to proceed with accurate and minimally invasive orthodontic traction in order to recover a mandibular canine.

## 1. Introduction

Dental impaction represents a common issue of orthodontic and surgical interest [1]. Despite the extensive literature concerning impacted maxillary canines, few articles exist which report on mandibular canine impaction and the treatment available. The reasons are related both to a lower mandibular canine impaction prevalence (between 0.3% and 2.8%) [2,3,4,5] and to the lower chances of orthodontic recovery [6].

The incidence of impacted mandibular canines is predominantly unilateral, with no gender difference [7]. This anomaly is related to aetiological mechanical or genetic factors such as abnormal deciduous canine resorption or eruptive disturbances, severe space discrepancies, narrow arches and delayed eruption [2]. Many impacted mandibular canines are displaced midline, causing transmigration [8,9]. 

Four options for treating this condition have been reported in the literature: radiographic monitoring, surgical extraction, orthodontic traction and autotransplant [2,3,10].

Nevertheless, it is the responsibility of the orthodontist in collaboration with the oral surgeon to attempt the recovery of the element in the arch and its proper repositioning to achieve ideal function and aesthetics, where deemed possible and advantageous [11,12]. Indications for the treatment of the impacted mandibular canine depend on the age of the patient, the occlusion and the degree of crowding, the stage of development of the canine root, the position of the canine, and the relationship between the canine and the adjacent teeth [12,13]. It should be considered that canine impaction is generally diagnosed when its root is already well developed; this can complicate orthodontic traction and correct repositioning in the dental arch. If an ectopic canine is detected early, a simple, less painful and more effective preventive treatment can be provided [14]. With timely interception and surgical exposure with orthodontic traction, impacted mandibular canines can be brought into an ideal position in the dental arch [2]. The frequently unfavourable position until transmigration and the greater bone density of the mandible [15] make the traction of impacted mandibular canines more complex than the corresponding impacted maxillary canines. Temporary anchorage devices (TADs) in orthodontic traction, such as miniscrews, provide a stable source of anchorage and help to reduce the negative effects on other teeth [15,16]. Few clinical studies have evaluated the orthodontic traction of the impacted mandibular canine using a skeletal anchorage [17,18]. This case report shows a digital workflow for the management of an impacted mandibular canine with the aid of Computer Aided Design/Computed-Aided Manufacturing (CAD/CAM) technology and TADs.

## 2. Case Report

A case of an 11-year-old subject in early mixed dentition with an impacted mandibular canine treated for orthodontic disimpaction with a skeletal anchored custom-made device is reported. 

### 2.1. Diagnosis and Aetiology

The 11-year-old subject attended the first orthodontic appointment in the Orthodontic Unit of the Department of Oral and Maxillo-Facial Sciences, Sapienza, University of Rome, Italy.

In the medical history, the parents report no previous illnesses or surgeries. The patient is in good general health.

**Figure 1:**
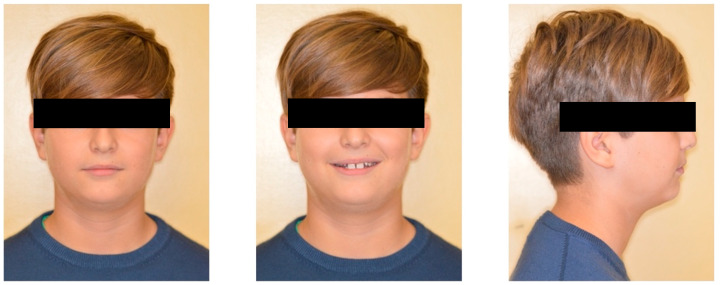
Pre-treatment extraoral photographs.

Extraoral examination revealed an oval-shaped face, proportional and symmetric, with lip competence but an increased exposure of the upper incisors while smiling. The facial profile was straight and the nasolabial angle appeared to be increased (Figure 1).

**Figure 2:**
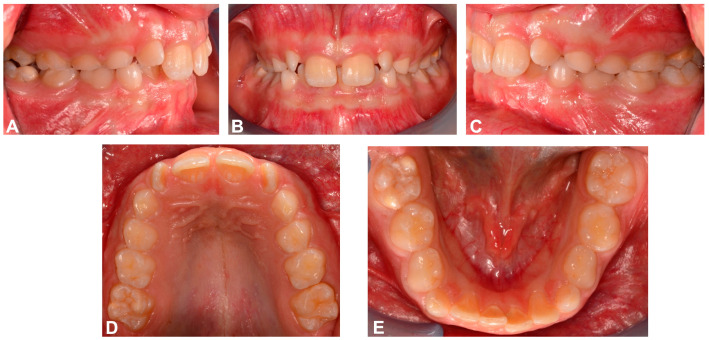
Pre-treatment intraoral photographs. (**A**) Right lateral intraoral view; (**B**) frontal intraoral view; (**C**) left lateral intraoral view; (**D**) upper occlusal intraoral view; (**E**) lower occlusal intraoral view.

Intraoral analysis revealed an early mixed dentition, Class I molar and canine relationships on both sides and mild anterior crowding in the upper and lower arch, with elements 1.2 and 2.2 rotated. Overjet and overbite were increased, the upper midline was centred in relation to the facial midline and the lower midline was not coincident with the upper midline but deviated 1.5 mm to the right. The maxillary arch showed reduced transverse dimensions and there was a molar crossbite on the right side (Figure 2).

**Figure 3:**
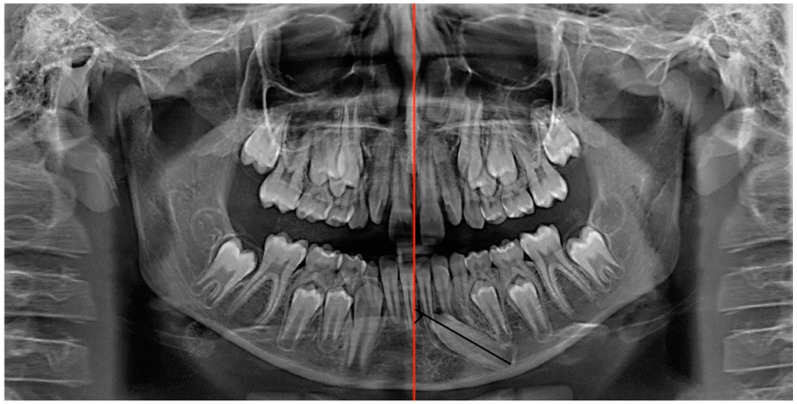
Pre-treatment panoramic radiograph. The angle subtended by the line passing through the mandibular midline (red) and the line passing through the long axis of the mandibular canine (black) were traced.

A panoramic radiograph showed that element 3.3 was deviated from its normal eruption path. The cuspid of the impacted canine was tilted mesially and positioned at the level of the right central incisor (element 3.1), close to the lower third of the root. The angle of the long axis of the canine to the line passing through the midline, traced according to Bertl et al. [4], was 60°. The deciduous element 7.3 was still present with its entire root (Figure 3).

**Figure 4:**
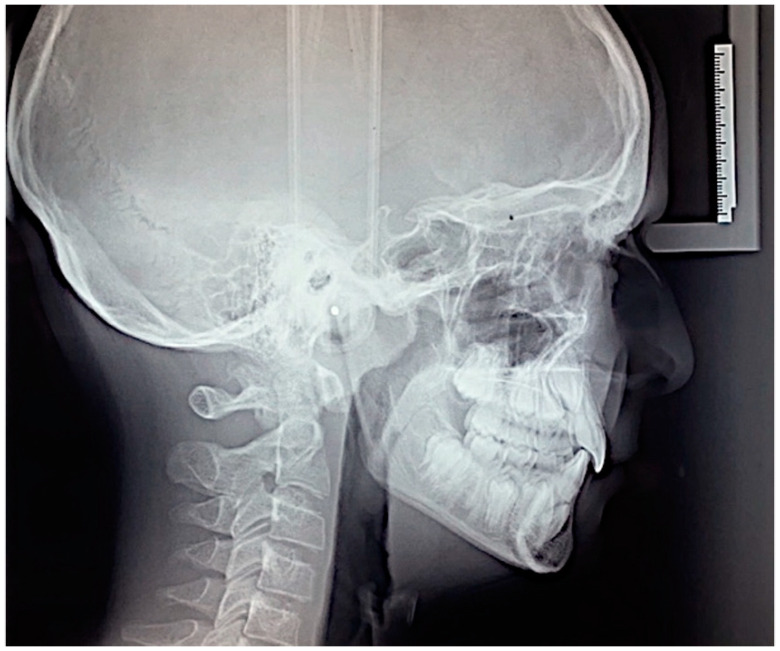
Pre-treatment lateral cephalogram.

The cephalometric analysis performed using Oris Ceph software Rx CE (Oris Ceph, Elit Computer, Vimodrone, Milan, Italy) showed a skeletal Class I relationship (skeletal class reference angle formed by maxillary point A, nasion and mandibular point B; ANB = 2°) with a hypodivergent vertical grow pattern (angle of divergence from the Frankfurt plane and the mandibular plane, FMA = 21°). The maxillary incisors were palatally inclined (angle of the maxillary incisor inclination with the palatal plane, U1-PP = 102°) and the angle of the mandibular incisors was in in the normal range (angle of inclination of mandibular incisor with mandibular plane, IMPA = 89°) (Figure 4).

A second-level radiographic examination was required to assess the relationship of the mandibular canine with its neighbouring elements. Root resorption of adjacent teeth was absent, and the vestibular side of the impacted tooth was confirmed.

### 2.2. Treatment Objectives

The primary objective was the recovery and repositioning of the impacted mandibular canine. A secondary objective was the dentoalveolar correction of the transverse contraction of the maxilla.

### 2.3. Treatment Alternatives

Different alternative treatment options for mandibular canine disimpaction were considered. 

One option was the extraction of the ipsilateral deciduous canine, followed by waiting for the spontaneous eruption of the element; this option was not considered effective due to the risk of possible root resorption of adjacent teeth considering the presence of “vis-a-tergo” of element 3.3.

Another alternative option was the extraction of element 3.3 and the retention of the primary canine until its exfoliation. This treatment option was considered invasive and irreversible and was not considered by the subject.

The last option was orthodontic traction. However, the distal surface of the crown of the lower first molar was partially covered by gingiva and did not allow a good fit of the band of a dental anchorage disinclusion appliance. Moreover, the same traction in the mandible was made more difficult by the higher density of the jaw bone compared to the maxilla [16], so traction by means of dental anchorage was not considered the best alternative. 

It was decided in agreement with the subject’s guardians to proceed with the disimpaction of the tooth element by means of a custom-made appliance to be skeletally anchored.

Contextually, the quad-helix appliance was used to resolve the dento-alveolar contraction of the maxilla.

### 2.4. Treatment Progress

**Figure 5:**
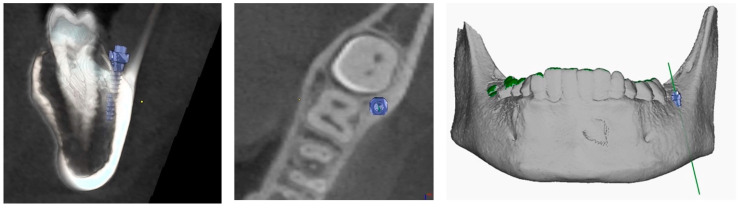
Planning of miniscrew insertion on CBCT (Cone Beam Computed Tomography) from different views.

**Figure 6:**
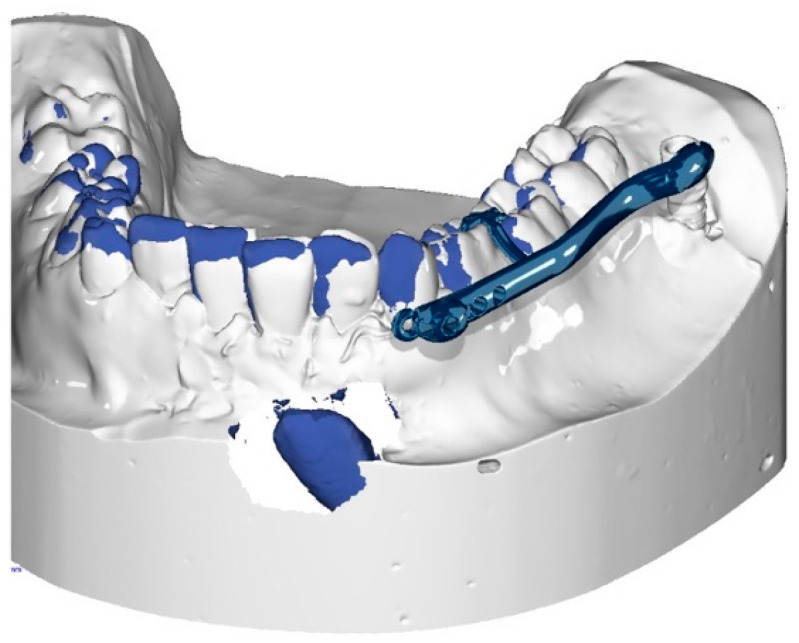
Planning of the customized skeletally anchored device in the three-dimensional (3D) digital model of the lower arch.

**Figure 7:**
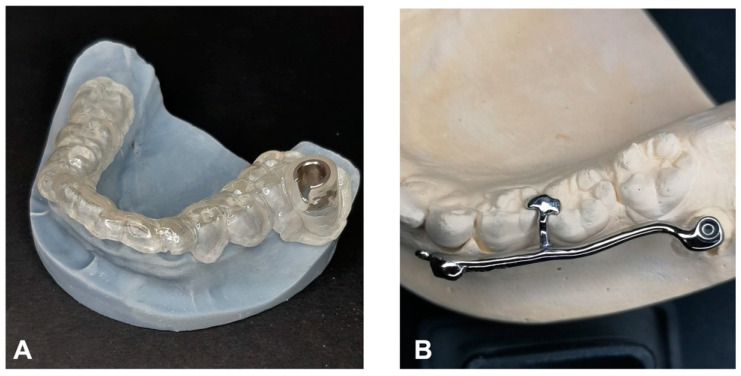
Customized surgical template (**A**) and disimpaction device with miniscrews (**B**) on the 3D-printed digital mandible model.

The procedure and potential risks associated with canine disimpaction, with particular emphasis on the risk of root resorption to adjacent elements, were fully explained to the subject and parents and signed informed consent for the publication of images and data was obtained. The disimpaction treatment consisted of a surgical phase of exposure of the impacted canine, with the possible removal of eruptive obstructions, and an orthodontic traction phase performed with a custom-made skeletal and dental anchorage device. The miniscrew insertion sites were planned on 3D images generated by merging CBCT and digital dental model images. The TADmatch 3D module of the Onyxceph3™ software (Image Instruments, Chemnitz, Germany) was used for the computer-guided planning of screw insertion using a surgical guide (Figure 5). A CAD/CAM digital workflow was performed to design the surgical guide and the skeletal anchoring disimpaction device using the Ortho Apps 3D module of the Onyxceph3™ software 3.2.180 Build 492–K2 (Image Instruments, Chemnitz, Germany) (Figure 6). The surgical guides were fabricated using TruPrint 1000 (TRUMPF Homberger S.r.l, Buccinasco, Italy) and the disimpaction device by laser melting technology with cobalt chrome metal powder (Stratasys OrhoDesktop; Stratasys, Rehovot, Israel) (Figure 7).

The length and diameter of the self-drilling titanium miniscrews used (BENEfit, PSM medical solutions, Tuttlingen, Germany) were predetermined during the planning phase based on bone anatomy (length: 13 mm; diameter: 2.3 mm). The miniscrew was inserted in the buccal shelf of the mandible, at the level of the mandibular first molar, on the left side; the appliance extended buccally from the miniscrew until the crown of the deciduous canine with a terminal hook and progressively distal holes for the insertion of orthodontic traction. Given the distance of the miniscrew from the disimpaction site, the device was designed to be very rigid with steel alloy with the rest cemented to the dental elements, which also had the function of preventing the device from rotating given that miniscrews are not equipped with effective anti-rotation devices. 

#### 2.4.1. Surgery

The subject underwent a routine medical examination to assess suitability for surgery and began antibiotic therapy one hour prior to surgery to minimize the risk of bacterial contamination.

The subject’s mouth was rinsed with a 0.2% chlorhexidine solution prior to the surgical exposure of the impacted canine and the insertion of the miniscrews. Local anaesthesia was given with 2% carbocaine with adrenaline in the ratio 1:100,000.

**Figure 8:**
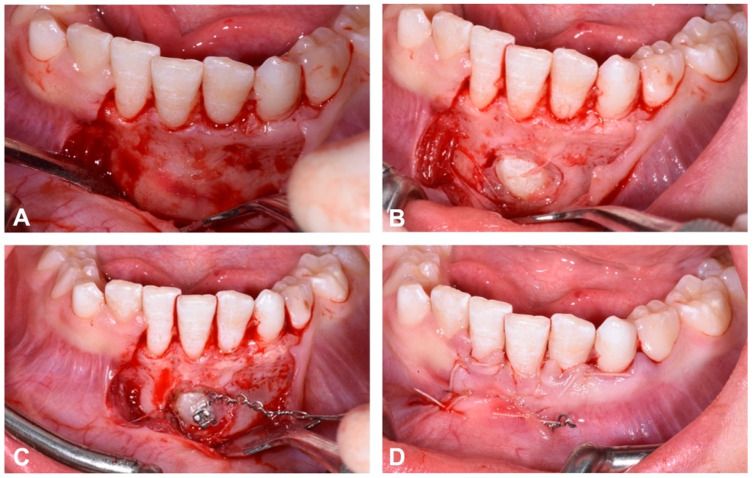
Flap opening (**A**); surgical exposure of the buccal side of impacted crown’s canine (**B**); positioning of the metal bracket for orthodontic traction (**C**); flap repositioned with resorbable suture and visible traction ligature (**D**).

The surgical exposure of the affected canine was performed with a full thickness flap. A bone incision was then made at the level of the cusp of the canine until two thirds of the crown was exposed. A bracket, to which a metal chain with traction loops had previously been welded, was applied using an adhesive technique. The visible part of the tooth crown was first etched with 37% phosphoric acid and the bracket was bonded with a light-curing adhesive system (Transbond XT, 3M Unitek, Monrovia, CA, USA). At the end of the surgical procedure, the flap was repositioned with a resorbable Nytilon 4.0 suture and one eyelet of the ligature was left on the surface to be used for overhead traction (Figure 8).

The use of the surgical guide, to ensure minimally invasive TAD insertion, improved the primary stability and reduced the risk of injury to contiguous anatomical structures.

The tooth-supported surgical guide was also used to create the guide hole (pre-drilling). A self-drilling miniscrew was picked up with the screwholder of a contra-angled handpiece and inserted by cordless screwdriver with a torque calibration system (ISD900, NSK, Tochigi, Japan). The pre-drilling phase was performed with the same cordless contra-angled handpiece with a drill (1.8 × 33 mm WL 15 mm). During the pre-drilling phase, the water jet was used to cool the drill in contact with the bone at a speed of 550 rpm. The miniscrew insertion torque was set at 40 N cm and a rotational speed of 25 rpm. It was not necessary to use a coolant. The screwholder was equipped with a stop to allow the desired insertion depth. The screwholder was adapted perfectly to the master tube inserted into the guide hole of the surgical template.

Once the miniscrew was in place, its primary stability was assessed, which was a prerequisite for connecting the custom device. The primary stability was assessed by considering the proportional relationship with the insertion torque [19].

**Figure 9:**
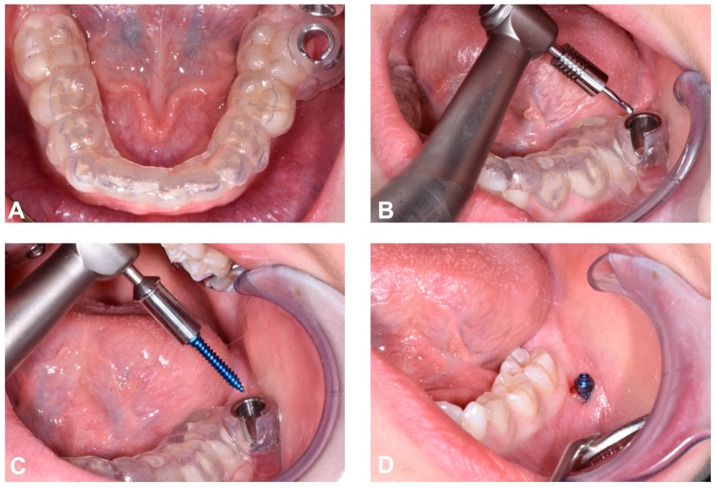
Intraoral occlusal view of surgical guide insertion (**A**); pre-drilling to guide miniscrew insertion (**B**); miniscrew insertion (**C**); miniscrew in place (**D**).

The device was then connected to the miniscrew using a fixation screw system and the dental rest was cemented to the primary teeth (Figure 9).

#### 2.4.2. Orthodontic Traction

**Figure 10:**
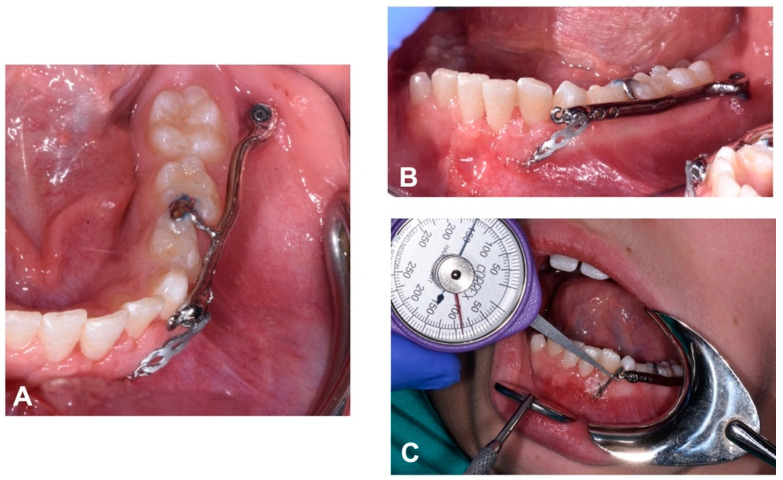
Occlusal (**A**) and lateral (**B**) intra-oral view of orthodontic traction with an elastic chain and its activation with the aid of the dynamometer (**C**).

The traction was activated on the same day, immediately after surgery. An elastic module was attached to the metal chain welded to the supplied canine button. The elastic force was calibrated to 150 g using a manual dynamometer Correx, 25–250 g (Haag-Streit Diagnostics, Koeniz, Switzerland). The elastic traction was then connected to the device by another metal ligature, which secured it to the hole furthest from the impaction site (Figure 10).

Every 20 days, the traction was reactivated by replacing the elastic module and attaching it to the loop closest to the impaction site.

**Figure 11:**
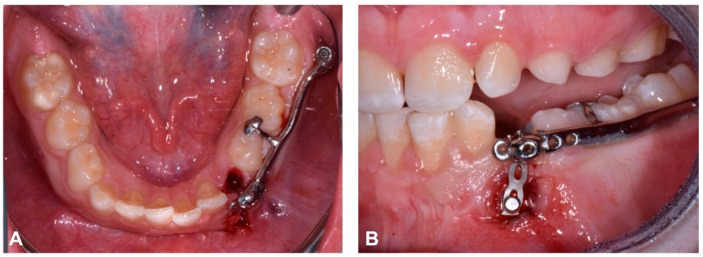
Deciduous extraction (7.3) (**A**) and gingivectomy to move bracket apically and reactivate traction on impacted canine (**B**).

**Figure 12:**
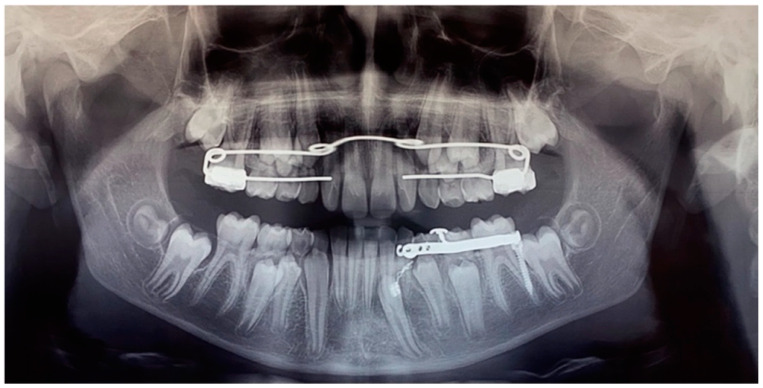
Panoramic radiography of treatment progress (1 year after the first panoramic radiograph).

When the bracket attached to the traction element was visible through the gingiva after 8 months, the ipsilateral deciduous canine (element 7.3) was extracted and a gingivectomy was performed to place the button more apically on the crown to continue the traction (Figure 11 and Figure 12). 

### 2.5. Treatment Results

**Figure 13:**
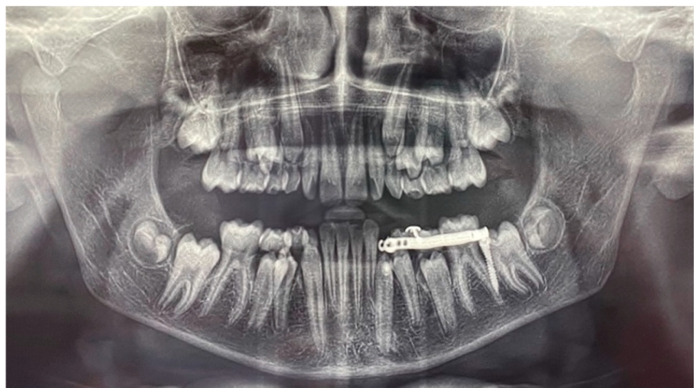
Panoramic radiograph at the end of the orthodontic traction with the customized skeletal anchorage device.

After 12 months, the mandibular canine (element 3.3) was disimpacted, in the axis with its eruptive path and with a correct eruption angle (Figure 13). The crown of the element was visible. The space present and the subject’s still-early mixed dentition was considered favourable to spontaneous eruption and the tooth was allowed to passively erupt. The disimpaction appliance was then removed and the miniscrew, still stable, was removed by unscrewing it.

**Figure 14:**
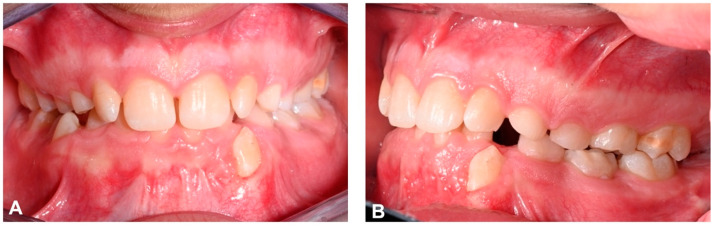
Frontal (**A**) and lateral (**B**) intraoral view after removal of the customized disimpaction device and miniscrew.

The tooth element was followed over time through clinical monitoring every month and radiographic follow-up every 6 months (Figure 14).

## 3. Discussion

From a therapeutic point of view, the time of diagnosis of an impacted tooth plays a crucial role in treatment options and prognosis. The early diagnosis of ectopically positioned mandibular canines is important to allow interceptive treatment. Over the years, several treatment options, such as surgical removal, exposure and orthodontic alignment, transplantation and observation, have been reported in the literature for the management of this condition [2]. The most common treatment strategy for impacted mandibular canines has been surgical removal, as this has been considered easier and quicker than repositioning the tooth. However, it is important to consider that the waiting time for a prosthetic replacement of the permanent canine could be long, with consequent aesthetic and functional discomfort for a subject who is still growing [10].

Orthodontic treatment is indicated when there is sufficient space to align an impacted mandibular canine, or when this can be achieved orthodontically, and the tooth is at a favourable stage of root development [20]. In the present case, the subject’s age, stage of root development, position within the bone, angle and depth of the impacted tooth were all decisive reasons for choosing orthodontic traction as the treatment option. 

The use of traditional appliance traction was not chosen. In a recent study, with the aim of using controlled light forces, two different dental anchorage traction devices were applied [21]. In our case, effective anchorage and force control was achieved by using only a single-sided device. In another case report of bilateral disimpaction, a customized tooth-supported anchorage devices cemented with bands to a skeletal anchorage traction device was preferred to avoid the risk of TAD insertion on teeth and on periodontal tissue [22]. Regarding this, in order to avoid damage to adjacent structures, in our case the miniscrew was computer-guided into position in the buccal shelf and our device was simpler and easier to activate [23].

Only two studies in the literature have documented the use of TADs for orthodontic traction of mandibular impacted canines [17,18]. In the present case, in contrast to previous reports where interradicular miniscrews were used, the miniscrew was inserted into the buccal shelf as an active anchorage. The placement of the miniscrew was computer-guided and provided stability to the system, greater comfort for the subject and ease of use for the clinician. 

The use of buccal shelf TADs eliminates adverse dental and periodontal effects [24] on anchorage elements during lower canine disimpaction, causes no discomfort or pain during placement and treatment and is compliance-free [25]. CAD/CAM digital technology, combined with 3D printing, enables the creation of a surgical guide for positioning the miniscrew and the customized devices used for disimpaction with greater accuracy and predictability, ensuring high-quality bone support [26,27,28].

Although orthodontic traction is the most complex therapeutic choice to implement, accurate and minimally invasive orthodontic traction can be made possible with the help of CAD/CAM technology.

## 4. Conclusions

The treatment of impacted mandibular canines with orthodontic traction represents a challenge to the clinician. Although treatment times can be long, successful disimpaction can be achieved with appropriate biomechanics, facilitated by CAD/CAM technology, and with the aid of a customized, miniscrew-supported appliance to minimize or avoid adverse effects and achieve the best results in rehabilitating an important element such as the mandibular canine.

## Data Availability

No new data were created or analyzed in this study. Data sharing is not applicable to this article.
